# A straightforward method to compute average stochastic oscillations from data samples

**DOI:** 10.1186/s12859-015-0765-z

**Published:** 2015-10-19

**Authors:** Jorge Júlvez

**Affiliations:** 1 Cambridge Systems Biology Centre, University of Cambridge, Tennis Court RoadCB2 1GA, Cambridge, UK; 2 Department of Computer Science and Systems Engineering, University of Zaragoza, María de Luna 1, Zaragoza, 50018 Spain

**Keywords:** Population dynamics, Stochastic oscillations, Average behavior, Stochastic processes, Jump Markov processes

## Abstract

**Background:**

Many biological systems exhibit sustained stochastic oscillations in their steady state. Assessing these oscillations is usually a challenging task due to the potential variability of the amplitude and frequency of the oscillations over time. As a result of this variability, when several stochastic replications are averaged, the oscillations are flattened and can be overlooked. This can easily lead to the erroneous conclusion that the system reaches a constant steady state.

**Results:**

This paper proposes a straightforward method to detect and asses stochastic oscillations. The basis of the method is in the use of polar coordinates for systems with two species, and cylindrical coordinates for systems with more than two species. By slightly modifying these coordinate systems, it is possible to compute the total angular distance run by the system and the average Euclidean distance to a reference point. This allows us to compute confidence intervals, both for the average angular speed and for the distance to a reference point, from a set of replications.

**Conclusions:**

The use of polar (or cylindrical) coordinates provides a new perspective of the system dynamics. The *mean* trajectory that can be obtained by averaging the usual cartesian coordinates of the samples informs about the trajectory of the *center of mass* of the replications. In contrast to such a *mean cartesian* trajectory, the *mean polar* trajectory can be used to compute the average *circular motion* of those replications, and therefore, can yield evidence about sustained steady state oscillations. Both, the coordinate transformation and the computation of confidence intervals, can be carried out efficiently. This results in an efficient method to evaluate stochastic oscillations.

## Background

Randomness plays a crucial role in the time evolution of most biological systems [1–3]. This implies that it is not possible to determine with absolute certainty how a given biological system will evolve in the future. Thus, one can at most aim at performing some statistical analyses to establish the probabilities of the potential future evolutions. This is true for molecular systems, where each molecule in the system is taken into account, and for population systems, where molecules and cells are disregarded, and just organisms are considered. This fact makes particularly difficult the study of oscillations, which are essential to understand biological systems as the circadian clock [4], epidemiological and ecological systems exhibiting non-seasonal fluctuations [5] (as measless or chicken pox [6, 7]), gene regulation networks where the expression levels of proteins fluctuate [8], etc.

Many biological systems are usually defined by a set of species and a set of reactions. At a given time instant, the state of the system is given by the number of individuals of each species, i.e., the state can be expressed as a vector of natural numbers in which each component of the vector is associated to a species. The occurrence of a reaction produces a change in the state of the system. As deducing the exact occurrence time of the reactions is nearly impossible, it is frequently assumed that they happen at random. This implies that many biological systems can be considered to be inherently *discrete* and *stochastic* [1, 9–11], and the dynamics of the populations involved in these systems can be appropriately modeled by means of jump Markov processes on the natural numbers [12].

The *master equation* associated to these processes determines accurately the variation over time of the probabilities of the potential states of the system. Unfortunately, the master equation suffers from the *curse of dimensionality* and is hardly ever analytically solvable [13]. Thus, alternative approaches must be sought to study the system dynamics.

A traditional approach consists in considering the *reaction rate equation* which is given by a system of Ordinary Differential Equations (ODE) [14–16]. Under some mild assumptions, the reaction rate equation determines the time evolution of the expected values of the system populations. The reaction rate equation has been intensively used to model and analyze systems in chemistry, biology and engineering, and offers the possibility to take advantage of the large existing toolbox for analysis purposes of dynamical systems described by differential equations [17]. This approach is also known as *continuous approximation*, *fluid approximation*, *mean field approximation* and *deterministic limit* [15, 18]. Notice that the state trajectories yielded by this approach are *continuous* and *deterministic*, while the system under study is *discrete* and *stochastic*. Although this approach provides accurate results for highly populated systems, it might fail at capturing important properties, as oscillations, commutations, and stochastic resonance in biological systems where the number of species is relatively low [5, 19, 20]. In such systems, stochasticity becomes increasingly important.

An alternative approach is not to solve exactly the master equation, which can be computationally prohibitive even in small systems, but to deal with its first order moments [13]. When the considered system is nonlinear, each central moment depends on a higher order moment what would lead to an infinite number of equations [21]. In order to avoid this problem, the Taylor expansion can be truncated, or moment closure techniques [22] can be applied. This approach is computationally efficient and usually performs well in practice. The limitations of the approach lie in the difficulty to determine the number of moments to consider, and the stiff and unstable equations that can be obtained for high number of moments for some systems, e.g., a prey-predator system like the one given by Lotka-Volterra equations.

Another approach is to consider drift and diffusion terms of continuous populations by means of *stochastic differential equations* [23, 24]. The inherent stochasticity of the system can now be captured and transient and steady state analysis can be carried out. Nevertheless, in this approach the mathematical model is still continuous what could lead to significant inaccuracies when the populations of the system are small.

Many dynamical properties of biological systems can be verified by means of probabilistic model checking [25, 26], a formal verification technique for stochastic systems. Based on the specification of a property in temporal logic, a model of the system is constructed in which each state represents a possible configuration of the system. Although model checking techniques have been proved successful in many application domains, they suffer from the state explosion problem inherent to many discrete systems.

The dynamics of a biological system can also be studied by stochastic simulation algorithms [27, 28]. These algorithms provide *exact samples or replications* of the evolution of the discrete stochastic system. Once several replications are computed, statistical analyses can be performed on the replications to extract quantitative information of interest, e.g., a confidence interval for the average population of a species in the steady state. Although stochastic simulation algorithms are increasingly efficient, they can be computationally expensive if small confidence intervals are required, as this usually requires a large number of samples.

This work proposes a straightforward procedure to assess stochastic oscillations of biological systems. More precisely, the goal of the procedure is to obtain confidence intervals both for the average angular speed and for the average distance to a given reference point. These confidence intervals are obtained from a set of replications obtained by a stochastic simulation algorithm. Consider several replications of a system exhibiting sustained stochastic oscillations. Given that the replications are stochastically out of phase, if the trajectories of the populations of the different replications are averaged, the resulting trajectory will show damped oscillations and will eventually converge to a point. In other words, oscillations cancel out in the averaged trajectory.

In order to overcome this problem, the procedure transforms the original cartesian coordinates of the computed samples, in which each dimension is associated to a species, to another coordinate system: polar coordinates for systems with two species [29], and cylindrical coordinates for systems with more than two species. In these coordinate systems, the angular coordinate will be computed in such a way that it represents the total angular distance run by the system, i.e., it is not constrained to the interval [0,2*π*). Then, instead of computing the cartesian average of populations to obtain confidence intervals, the polar (or cylindrical) average is considered. This way, the average circular motion of the system state around a given reference point (or axis) can be computed without being affected by the replications being stochastically out of phase. Hence, the use of a polar coordinate system can easily uncover stochastic oscillations in the replications otherwise hidden to the cartesian coordinate system.

Most of the existing methods to detect stochastic oscillations are based on the use of the power spectral density and the autocorrelation of the time series [30, 31]. Power spectra are, for instance, used in [32] to explore the relationship between noisy cycles and quasi-cycles, and in [33] to assess oscillations of quasi-cycles in discrete-time models. On the other hand, the autocorrelation function, together with marginal distributions of population sizes, is used in [34] to distinguish between noisy cycles and quasi-cycles, and in [35] to quantify the effect of noise on a periodic signal. An advantage of the method proposed here with respect to previous approaches is that it can detect oscillations in short time series. This is due to the fact that the method makes use of a description of the system dynamics in polar coordinates and, in consequence, it does not need to find repetitive patterns along the time series to detect an oscillation.

An alternative method, not based on the power spectrum, to evaluate oscillations consists of making use of probabilistic model checking [36]. This method can provide relevant quantitative information of oscillations, but has to deal with the state explosion problem. The method proposed here does not perform an exhaustive state space exploration, and hence, it does not suffer from the state explosion problem.

On the other hand, as discussed in the Methods section, the ODE given by the reaction rate equation (or deterministic limit) captures the evolution of the expected values of the cartesian coordinates of the system. Following the ideas presented above, an alternative ODE, namely ODE (11), can be designed to capture the evolution of the expected values of the polar coordinates. Such an ODE provides a different perspective of the system dynamics and can be used to quickly detect potential stochastic oscillations without the need of simulation.

The method is described in detailed in the Methods sections. It is applied on four different case studies in the Results and discussion section. The obtained average polar trajectories are compared to the average cartesian coordinates and to the deterministic trajectory yielded by the reaction rate equation.

## Methods

This section first introduces the notation for the system parameters and the ODE determining the deterministic limit of the system under consideration. Then, the assessment of the evolution of affine and quadratic functions by the deterministic limit is studied. Finally, the method to evaluate sustained oscillations in stochastic systems is presented.

### System parameters and deterministic limit

The following parameters are used to describe the system dynamics (in these definitions, *A*_*i*_ denotes the *i* ^*th*^ component of vector *A*).

- $q\in \mathbb {N}$ denotes the number of populations (or species).
- $n\in \mathbb {N}$ denotes the number of events (or reactions).
- $\mathbf {X}(t)\in \mathbb {N}^{q}_{\geq 0}$ is the state of the system at time *t* (*X*_*i*_(*t*) denotes the number of elements of population *i* at time *t*)
- $\nu \in \mathbb {N}^{q\times n}_{\geq 0}$ is the stoichiometry matrix, i.e., ${\nu _{i}^{j}}$ is the change produced in population *i* by event *j* (*ν* ^*j*^ will denote the *j* ^*th*^ column of *ν*, i.e., stoichiometry vector of reaction *j*, and *ν*_*i*_ will denote the *i* ^*th*^ row of *ν*).
- $V \in \mathbb {R}_{>0}$ is the size (or volume) of the system.
- $W_{j}:\mathbb {R}_{\geq 0}^{q}\times \mathbb {R}_{> 0}\rightarrow \mathbb {R}_{\geq 0}$ is the transition rate function, i.e, *W*_*j*_(**X**(*t*),*V*) is the rate associated to event *j* for population **X**(*t*) and system size *V*.

It is assumed that each transition rate function *W*_*j*_(**X**(*t*),*V*) is a differentiable nonnegative function that does not depend on time (for readability we will use **X** rather than **X**(*t*)). Further, following the notation in [20], it is assumed that *W*_*j*_(X,*V*) satisfies the density dependence condition, i.e., *W*_*j*_(**X**,*V*)=*V*·*w*_*j*_(**X**/*V*), where *w*_*j*_(**X**/*V*) is a nonnegative function of real arguments on the system densities. This condition states that if the densities are kept constant while the system size changes from *V* to *V* ^′^, then the transition rates change by a factor *V* ^′^/*V*. In the following, *W*_*j*_(**X**(*t*),*V*) is simplified to *W*_*j*_(**X**) for clarity, and densities will be expressed in lowercase, e.g., **x**=**X**/*V*.

The system is modeled as a jump Markov process in which events are exponentially distributed with rates *W*_*j*_(**X**). The occurrence of an event *j* changes the system state from **X** to **X**+*ν* ^*j*^. Given that all rates are exponentially distributed, the next event time is also exponentially distributed with rate $R(\mathbf {X})=\sum _{j=1}^{n} W_{j}(\mathbf {X})$, and the probability that the next event is event *j* is *W*_*j*_(**X**)/*R*(**X**).

Given a sample path of a jump Markov process, the *embedded process* is the sequence of consecutive states {**X** ^0^,**X** ^1^,**X** ^2^,…,**X** ^*k*^,…} of the path. From a sequence {**X** ^0^,**X** ^1^,**X** ^2^,…,**X** ^*k*^,…}, sample paths of the Markov process can be built by producing times for each event with exponentially distributed random variables.

**Deterministic limit:** Let $F_{i} = \sum _{j=1}^{n} {\nu _{i}^{j}} w_{j}(\mathbf {x})$ be the vector field for species *i*, and assume that $\sum _{j=1}^{n} |{\nu _{i}^{j}}| w_{j}(\text {x})<\infty $ and *F* is Lipschitz continuous, i.e., ∃*M*≥0 such that |*F*(**x**)−*F*(**y**)|≤*M*|**x**−**y**|. Then, the deterministic limit behaviour of the system when *V* tends to infinity is given by the following ODE [15, 16]: $\frac {dx_{i}}{dt}=F_{i}(\mathbf {x})=\sum _{j=1}^{n} {\nu _{i}^{j}} w_{j}(\mathbf {x})$. This ODE can be scaled by *V* to obtain a deterministic continuous trajectory for a system with size *V*:

(1) $$ \frac{dX_{i}}{dt}=\sum\limits_{j=1}^{n} {\nu_{i}^{j}} W_{j}(\mathbf{X})  $$

**Fig. 1:**
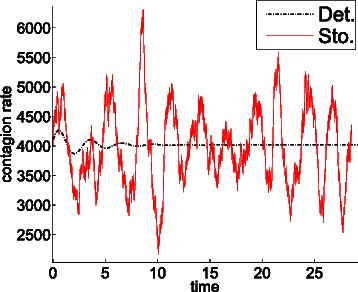
Deterministic and stochastic evolution of the contagion rate of the epidemic system. Evolution of the contagion rate according to ODE (1) (dotted line), and according to a single stochastic replication of the Markov process (solid line). In contrast to the deterministic evolution, the stochastic replication exhibits undamped oscillations

Under the conditions assumed in this section, the limit trajectory of the system densities, as the populations sizes tend to infinity, is obtained by the solution of ODE (1). Although such a *continuous* and *deterministic* trajectory can be meaningful in highly populated systems, it might disregard relevant phenomena caused by the inherent *discrete* and *stochastic* nature of biochemical systems. In fact, as the limit is never attained in practice, the trajectory yielded by ODE (1) must be used with caution when evaluating the time evolution of functions of interest, e.g., the evolution of the contagion rate in the epidemic system, see Fig. 1. In the following, it is shown that affine functions are appropriately evaluated by ODE (1), but more general functions, as quadratic functions used to measure distances, are not.

### Affine functions and quadratic functions

#### Affine functions

Let $f:\mathbb {R}_{\geq 0}^{q} \rightarrow \mathbb {R}$ be an affine function of the type *f*(**X**)=*A* **X**+*b* (for clarity, we avoid the use of transpose symbols). Let us first evaluate the change rate of *f* in the deterministic continuous trajectory provided by ODE (1). By the chain rule and (1), the total derivative of *f* with respect to time is:

(2) $$   \begin{aligned} \frac{d f}{d t} =\sum\limits_{i=1}^{q} \frac{\partial f}{\partial X_{i}}\frac{d X_{i}}{d t} & =\sum\limits_{i=1}^{q} \frac{\partial f}{\partial X_{i}}\left(\sum\limits_{j=1}^{n} {\nu_{i}^{j}} W_{j}(\mathbf{X})\right) \\ & =\sum\limits_{i=1}^{q} A_{i} \left(\sum\limits_{j=1}^{n} {\nu_{i}^{j}} W_{j}(\mathbf{X})\right) = A\nu W(\mathbf{X}) \end{aligned}  $$

where *A*_*i*_ is the *i* ^*th*^ element of vector *A*, and *W*(**X**) is a vector whose *j* ^*th*^ element is *W*_*j*_(**X**).

In order to estimate the speed of change of function *f* according to the Markov process, we will consider the expected increase of *f* produced by the occurrence of an event, and the average frequency of events. All the considered expected values are conditional on the current state. For brevity, $\mathbb {E}[\!\Delta f(\mathbf {X})|\mathbf {X}]$ is shortened to $\mathbb {E}[\!\Delta f(\mathbf {X})]$ to denote the expected increase of *f*, given **X**, of the embedded Markov process after the occurrence of an event (*Δ* is the usual finite difference operator). At a given state **X**, the expected increase of function *f* after the occurrence of an event is the weighted average of the increases of *f* produced by the different events:

(3) $$  \begin{aligned} \mathbb{E}\left[\Delta f(\mathbf{X})\right] &= \sum\limits_{j=1}^{n} \frac{W_{j}(\mathbf{X})}{R(\mathbf{X})}\left(\,f(\mathbf{X}{+}\nu^{j}){-}f(\mathbf{X})\right)\\ & =\sum\limits_{j=1}^{n} \frac{W_{j}(\mathbf{X})}{R(\mathbf{X})} \left(A(\mathbf{X}{+}\nu^{j}){+}b{-}(A\mathbf{X}{+}b)\right) \end{aligned}  $$

Given that at **X** the average number of events per time unit, i.e, frequency, is *R*(**X**), the average speed of change of *f* according to the Markov process can be approximated as:

(4) $$   \begin{aligned} R(\mathbf{X}) \mathbb{E}\left[\Delta f(\mathbf{X})\right] & =\sum\limits_{j=1}^{n} W_{j}(\mathbf{X}) \left(A(\mathbf{X} + \nu^{j}) + b - (A\mathbf{X} + b)\right) \\ & =\sum\limits_{j=1}^{n} W_{j}(\mathbf{X}) \left(\sum\limits_{i=1}^{q} A_{i} {\nu_{i}^{j}} \right)\\ & = \sum\limits_{j=1}^{n} \sum\limits_{i=1}^{q} A_{i} {\nu_{i}^{j}} W_{j}(\mathbf{X}) {=} A\nu W(\mathbf{X}) \end{aligned}  $$

Notice that the above equations are essentially the ones that the Dynkin formula [24] would produce. Hence, the affine function *f* is evaluated equally by ODE (1) and the Markov process (see (2) and (4)). Nevertheless, this is not the case for a more general function *f*.

#### Quadratic functions

Let *f* be the product of two affine functions *h*(**X**)=*C* **X**+*u* and *g*(**X**)=*D* **X**+*v*, i.e., *f*(**X**)=*h*(**X**)*g*(**X**)=(*C* **X**)(*D* **X**)+*C* **X** *v*+*D* **X** *u*+*u* *v*. The sum *C* **X** *v*+*D* **X** *u*+*u* *v* is an affine function and will be equally evaluated by ODE (1) and the Markov process, thus, to simplify the presentation we will assume that *u*=*v*=0 and hence, *f*(**X**)=(*C* **X**)(*D* **X**). By using (1), and given that *f* is a product of functions, the total derivative of *f* is:

(5) $$  {\fontsize{9.1}{6}\begin{aligned} \frac{d\ f}{d t} &= \frac{d\ hg}{d t} = h\frac{dg}{dt} + g\frac{dh}{dt} =h\sum\limits_{i=1}^{q} \frac{\partial g}{\partial X_{i}}\frac{dX_{i}}{dt} + g\sum\limits_{i=1}^{q} \frac{\partial h}{\partial X_{i}}\frac{dX_{i}}{dt}\\ & =\sum\limits_{i=1}^{q} \frac{dX_{i}}{dt} \left(h \frac{\partial g}{\partial X_{i}} + g \frac{\partial h}{\partial X_{i}}\right)\\ &=\sum\limits_{i=1}^{q} \sum\limits_{j=1}^{n} {\nu_{i}^{j}}W_{j}(\mathbf{X})(C\mathbf{X} D_{i} + D\mathbf{X} C_{i}) \\ & =((C\mathbf{X}) D+(D\mathbf{X}) C)\sum\limits_{j=1}^{n} \nu^{j}W_{j}(\mathbf{X}) \end{aligned}}  $$

As for affine functions, the average speed of change of *f* can be approximated by the expected increase of the Markov process, $\mathbb {E}[\!\Delta f(\textbf {X})]=\mathbb {E}[\!\Delta ((C\textbf {X})(D\textbf {X}))]$, times the average number of events per time unit, *R*(**X**). By the product rule of the finite difference operator (the product rule of the finite difference operator states: *Δ*(*h* *g*)=*h* *Δ* *g*+*g* *Δ* *h*+*Δ* *h* *Δ* *g*) and given that the vector of expected increases of populations is $\mathbb {E}[\!\Delta \mathbf {X}]{=}\sum \limits _{j=1}^{n} \nu ^{j}\frac {W_{j}(\mathbf {X})}{R(\mathbf {X})}$, the following equality is produced:

(6) $$ {\fontsize{7.5}{6}\begin{aligned} R(\mathbf{X})&\mathbb{E}\left[\Delta((C\mathbf{X})(D\mathbf{X}))\right]\\ &=R(\mathbf{X})\mathbb{E}[(C\mathbf{X})\Delta(D\mathbf{X})+(D\mathbf{X})\Delta(C\mathbf{X})+\Delta(C\mathbf{X})\Delta(D\mathbf{X})] \\ & =R(\mathbf{X})(C\mathbf{X}) D\mathbb{E}[\!\Delta\mathbf{X}]\,+\, R(\mathbf{X})(D\mathbf{X}) C\mathbb{E}[\Delta\mathbf{X}] +\,R(\mathbf{X})\mathbb{E}[\!\Delta(C\mathbf{X})\Delta(D\mathbf{X})] \\ & =(C\mathbf{X}) D\sum_{j=1}^{n} \nu^{j}W_{j}(\mathbf{X})+(D\mathbf{X}) C\sum_{j=1}^{n} \nu^{j}W_{j}(\mathbf{X}) +R(\mathbf{X})\mathbb{E}[\!\Delta(C\mathbf{X})\Delta(D\mathbf{X})] \\ & =((C\mathbf{X}) D+(D\mathbf{X}) C)\sum_{j=1}^{n} \nu^{j}W_{j}(\mathbf{X}) + R(\mathbf{X})\mathbb{E}[\!\Delta(C\mathbf{X})\Delta(D\mathbf{X})] \end{aligned}}  $$

From (5) and (6), the following equality showing the different speeds of change resulting from the ODE (1) and the Markov process is derived:

$$R(\mathbf{X})\mathbb{E}[\!\Delta((C\mathbf{X})(D\mathbf{X}))]=\frac{d\ f}{d t}\,+\,R(\mathbf{X})\mathbb{E}[\!\Delta(C\mathbf{X})\Delta(D\mathbf{X})] $$

Thus, *f* is, in general, evaluated differently by the ODE that represents the deterministic limit and the Markov process. Quadratic functions as *f*(**X**)=(*C* **X**)(*D* **X**)+*C* **X** *v*+*D* **X** *u*+*u* *v* appear naturally when estimating the evolution of certain reaction rates (as the contagion rate in the epidemic system in the Results and discussion section), the product of populations that could activate other events, or the squared distance to a given point. For this last case, it can be shown that the deterministic limit underestimates the speed of change of the squared distance with respect to a point *a* with coordinates (*a*_1_,…,*a*_*q*_). Let $L_{a}(\textbf {X})=\sum _{i=1}^{q}(X_{i}-a_{i})^{2}$, then, the speed of change of *L*_*a*_ provided by ODE (1) is:

(7) $$ \begin{aligned}  \frac{d L_{a}}{d t}=\sum\limits_{i=1}^{q} \frac{\partial L_{a}}{\partial X_{i}}\frac{d X_{i}}{d t} = \sum\limits_{i=1}^{q} 2(X_{i}{-}a_{i})\left(\sum\limits_{j=1}^{n} {\nu_{i}^{j}}W_{j}(\mathbf{X})\right) \end{aligned}  $$

On the other hand, by the chain rule *Δ*(*z* ^2^)=2*z* *Δ* *z*+(*Δ* *z*)^2^ and given that the expected increase of population *i* is $\mathbb {E}[\!\Delta \textbf {X}_{i}]{=}\sum \limits _{j=1}^{n} {\nu _{i}^{j}}\frac {W_{j}(\textbf {X})}{R(\textbf {X})}$, the speed of change of *L*_*a*_ estimated by the Markov process is:

(8) $$  \begin{aligned} R(\mathbf{X})\mathbb{E}[\!\Delta L_{a}(\mathbf{X})]&=R(\mathbf{X})\mathbb{E}\left[\Delta \sum\limits_{i=1}^{q} (X_{i}-a_{i})^{2}\right]\\ &= R(\mathbf{X})\sum\limits_{i=1}^{q} \mathbb{E}\left[\Delta (X_{i}-a_{i})^{2}\right] \\ & = R(\mathbf{X})\sum\limits_{i=1}^{q} \mathbb{E}\left[2(X_{i}-a_{i})\Delta X_{i}+(\Delta X_{i})^{2}\right] \\ & = R(\mathbf{X})\sum\limits_{i=1}^{q} 2(X_{i}-a_{i})\mathbb{E}[\!\Delta X_{i}]\\&\quad+R(\mathbf{X})\sum\limits_{i=1}^{q}\mathbb{E}\left[(\Delta X_{i})^{2}\right] \\ & = \sum\limits_{i=1}^{q} 2(X_{i}-a_{i})\left(\sum\limits_{j=1}^{n} {\nu_{i}^{j}}W_{j}(\textbf{X})\right)\\&\quad+R(\textbf{X})\sum\limits_{i=1}^{q}\mathbb{E}\left[(\Delta X_{i})^{2}\right] \end{aligned}  $$

From (7) and (8), the following equality is obtained:

(9) $$  R(\mathbf{X})\mathbb{E}\left[\Delta L_{a}(\mathbf{X})\right]= \frac{d L_{a}}{d t}+R(\mathbf{X})\sum\limits_{i=1}^{q}\mathbb{E}\left[(\Delta X_{i})^{2}\right]  $$

Thus, given that $\sum _{i=1}^{q}\mathbb {E}[\!(\Delta X_{i})^{2}]\geq 0$, the Markov process estimates that the system moves away faster from (or approaches slower) point *a* as long as events happen, i.e., as long as *R*(X)>0. In particular, if *a* is a fixed point it holds that $\frac {d L_{a}(a)}{d t}=0$, i.e., the effect of all the events cancels out, and hence, the trajectory given by the deterministic limit stays constant at *a*. However, according to the Markov process events will keep on occurring if *a* is not an extinction point, and hence, the system can move away from *a* at an average speed of $R(\textbf {X})\sum _{i=1}^{q}\mathbb {E}[(\Delta X_{i})^{2}]$ and describe a different trajectory.

Notice that as *V* increases and tends to infinity while the concentrations are kept constant, the trajectory of the jump Markov process will converge to that of the deterministic limit [15, 16]. Nevertheless, in many systems of interest, the value of *V* cannot be taken as infinity and just considering the deterministic limit can overlook important properties of the system dynamics.

### Systems with two species

By using similar mathematical developments as in the previous subsection, the expected value of the embedded process can be used to provide a different view (polar instead of cartesian) of the system evolution. Such a view is the result of estimating the distance and angle of the state of the system to a given reference point. The focus of this subsection is on systems with two species, i.e., *q*=2.

The trajectory yielded by ODE (1) in the phase space is tangent to the weighted average of the vectors *ν* ^*j*^ according to their transition rates at each time instant. That is, the future positions of the system are computed according to the weighted average of the cartesian coordinates of the vectors *ν* ^*j*^. As the size *V* tends to infinity, the evolution of the Markov process approaches the deterministic limit ODE (1). An alternative way to study the evolution of a process with size *V* is to perform a number of replications (or sample paths) and compute the mean populations at given sampling times.

Notice that, when computing the mean populations, one is averaging the cartesian coordinates. One can, however, consider other values to analyze the dynamical behaviour of the process. For instance, one could average the distance of the state to a given reference point. This average distance together with angular information with respect to the reference point would state the basis to extract dynamical information of the process in polar coordinates.

**Fig. 2:**
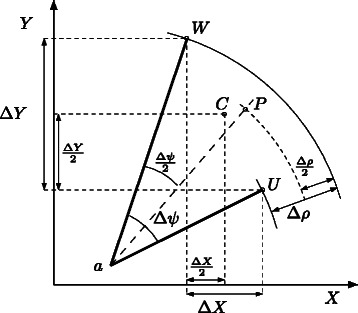
Average cartesian (point *C*) and polar (point *P*) coordinates of points *U* and *W* with respect to reference point *a*. *Δ* *X* and *Δ* *Y* are the increments in cartesian coordinates; *Δ* *ρ* and *Δ* *ψ* are the increments in distance and angle. These two averages provide different perspectives about the dynamics of the stochastic system

Assume that two replications of a given system have been performed, and one desires to estimate the average system trajectory over time. A common approach would be to compute the mean values of the populations at same time instants. Assume that at a given time instant *τ*, the number of individuals of the first species *X* given by the first replication is *U*_*x*_ and the number of individuals of the second species *Y* is *U*_*y*_ (Fig. 2). Let us also assume that the number of individuals given by the second replication at time *τ* is *W*_*x*_ and *W*_*y*_. The mean of these *cartesian* coordinates yields the *average* state *C*. Nevertheless, one might desire to evaluate, not the average cartesian coordinate, but the average position with respect to a given reference point. Figure 2 shows how state *P* is obtained as the mean polar coordinates of states *U* (with cartesian coordinates (*U*_*x*_,*U*_*y*_)) and *W* (with cartesian coordinates (*W*_*x*_,*W*_*y*_)) with respect to reference point *a*: the polar coordinates of *P* are simply the mean polar coordinates, i.e., angle and distance, of the polar coordinates of *U* and *W*.

The procedure to compute the average polar coordinates of a number of replications could be stated as follows:

1. Assume that *M* replications have been performed and the trajectories have been resampled at same sampling times. Let $\left (\!{X^{0}_{q}},{Y^{0}_{q}}\!\right)$, $\left (\!{X^{1}_{q}},{Y^{1}_{q}}\right)$, …, $\left (\!{X^{k}_{q}},{Y^{k}_{q}}\right)$, … be the cartesian coordinates of replication *q* at sampling times 0,1,…,*k*,….
2. Let the origin of the polar coordinate system be the reference point *a* with cartesian coordinates (*a*_*x*_,*a*_*y*_).
3. Each $\left ({X^{k}_{q}},{Y^{k}_{q}}\right)$ can be transformed to polar coordinates $\left ({\rho ^{k}_{q}},{\phi ^{k}_{q}}\right)$ with origin at *a* by using: $~~~{\rho ^{k}_{q}}=\sqrt {\left ({X^{k}_{q}}-a_{x}\right)^{2}+\left ({Y^{k}_{q}}-a_{y}\right)^{2}}$, ${\phi ^{k}_{q}}=\text {atan}$ $\left ({Y^{k}_{q}}{-}a_{y},{X^{k}_{q}}{-}a_{x}\right)$ where $\text {atan}(y,x): \mathbb {R}{\times } \mathbb {R}\rightarrow \mathbb {R}$ is the arctangent of a point with cartesian coordinates (*x*,*y*) that takes into account the quadrant. We will assume that the range of atan(*y*,*x*) is (−*π*,*π*] and that atan(0,0)=0.

This straightforward transformation to polar coordinates poses a problem when averaging the angle *ϕ* of replications. Assume that at a given step *k*, the states of two trajectories *i* and *j* are on the left half plane defined by *a*_*x*_, i.e., ${X^{k}_{i}}<a_{x}$ and ${X^{k}_{j}}<a_{x}$, and ${Y^{k}_{i}}$ is slightly higher than *a*_*y*_ while ${Y^{k}_{j}}$ is slightly lower than *a*_*y*_. Thus, ${\phi ^{k}_{i}}$ will be positive and close to *π* while ${\phi ^{k}_{j}}$ will be negative and close to −*π*. Hence, the mean of ${\phi ^{k}_{i}}$ and ${\phi ^{k}_{i}}$ will be close to 0 what is not useful as average angle.

**Fig. 3:**
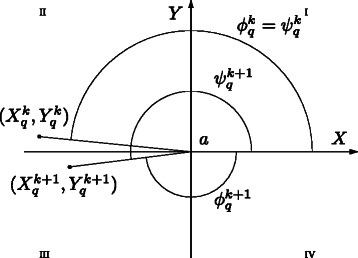
Polar average of points with angular coordinate close to *π*. The angular coordinates *ϕ* of the consecutive points $\left ({X_{q}^{k}},{Y_{q}^{k}}\right)$ and $\left (X_{q}^{k+1},Y_{q}^{k+1}\right)$ of the *q* ^*th*^ replication have opposite signs and absolute values close to *π*. As this can cause problems when averaging angles of different replications, the total angular distance run *ψ* is used instead of *ϕ*

In order to overcome this problem, we define a new value ${\psi ^{k}_{q}}$ to account for the overall angular distance run by the trajectory. Let us define ${\psi ^{0}_{q}}={\phi ^{0}_{q}}$, and for each *k*≥0, let us express ${\psi ^{k}_{q}}$ as ${\psi ^{k}_{q}}={z^{k}_{q}} 2 \pi +{h^{k}_{q}}$, with ${z^{k}_{q}}\in \mathbb {Z}$ and $-\pi <{h^{k}_{q}}\leq \pi $, i.e, ${z^{k}_{q}}$ is an integer representing the rounded number of completed loops and ${h^{k}_{q}}$ is the angular distance run on the current loop. The value of ${z^{k}_{q}}$ is positive(negative) if the angular distance was run anticlockwise(clockwise). In Fig. 3, state $\left (X_{i}^{k+1},Y_{i}^{k+1}\right)$ is reached after $\left ({X_{i}^{k}},{Y_{i}^{k}}\right)$, thus the total angular distance run at $\left (X_{i}^{k+1},Y_{i}^{k+1}\right)$ is $\psi _{q}^{k+1}$ and not $\phi _{q}^{k+1}$. Moreover, the number of completed loops at $\left (X_{i}^{k+1},Y_{i}^{k+1}\right)$ is $z^{k+1}_{q}=1$ as it was anticlockwise and $h^{k+1}_{q}$ is a negative value not lower than −*π*.

More formally, the value of ${\psi ^{k}_{q}}$ for *k*≥0 can be computed as follows:

(10) $$ {\fontsize{8.3}{8}\begin{aligned}{\psi^{k}_{q}}=\left\{ \begin{array}{ll} {\phi^{0}_{q}} & \text{if}\, k=0\\ z^{(k-1)}_{q} 2 \pi+{\phi^{k}_{q}}+2\pi & \text{if}\, k>0\, \text{and}\, h^{(k-1)}_{q}>\frac{\pi}{2}\, \text{and}\, {\phi^{k}_{q}}<-\frac{\pi}{2}\\ z^{(k-1)}_{q} 2 \pi+{\phi^{k}_{q}}-2\pi & \text{if}\, k>0\, \text{and}\, h^{(k-1)}_{q}<-\frac{\pi}{2}\, \text{and}\, {\phi^{k}_{q}}>\frac{\pi}{2}\\ z^{(k-1)}_{q} 2 \pi+{\phi^{k}_{q}} & \text{otherwise} \end{array} \right. \end{aligned}}  $$

where ${\phi ^{k}_{q}}=\text {atan}\big ({Y^{k}_{q}}{-}a_{y},{X^{k}_{q}}{-}a_{x}\big)$ for every *k*≥0. The first case of the above expression sets the initial value of ${\phi ^{0}_{q}}$. The second and third cases take into account the discontinuity of the angle returned by atan when the trajectory moves from the second to the third quadrant, and from the third to the second quadrant respectively. The forth case does not have to handle a change of quadrant and just computes the overall angular distance run by the system.

Thus, in the following the polar coordinates of the *q* ^*th*^ replication at the *k* ^*th*^ sampling time will be expressed as ${\big ({\rho ^{k}_{q}},{\psi ^{k}_{q}}\big)}$.

Once ${\psi ^{k}_{q}}$ is computed for every *k* and every replication, an average trajectory in polar coordinates can be obtained by computing the mean of ${\rho ^{k}_{q}}$ and ${\psi ^{k}_{q}}$ over all replications. The average polar trajectories reported in the Results and discussion section have been obtained by the described procedure.

The steps required to perform statistical analyses of steady state parameters of the polar trajectory are similar to the standard ones [37]. The focus will be on estimating the steady state mean values of the polar coordinates of the system within a given confidence interval with respect to a given reference point (*a*_*x*_,*a*_*y*_). In particular, (*a*_*x*_,*a*_*y*_) is taken as the average cartesian coordinates of the performed replications. It must be noticed that at the steady state of an oscillating system, the angular coordinate does not tend to a constant value but increases or decreases monotonically over time. In order to take into account this fact, instead of the mean angle *ψ*, the mean angular speed, which will be denoted as *ξ*, will be estimated. Given two parameters *α* and *Maxerr*, Algorithm 1 summarizes the tasks required to build *α* percent confidence intervals with *MaxErr* relative error for the mean distance and angular speed of the system with respect to (*a*_*x*_,*a*_*y*_).



As the interest is in estimating steady state means, the first step, (step 1), of Algorithm 1 is to determine the length of the transient state (or warm-up period) to make easier the initial-data deletion when computing averages. This task has been achieved by means of the *Welch’s procedure* [37, 38].

In order to compute confidence intervals that satisfy the requirements of the input parameters *α* and *MaxErr*, the *replication/deletion approach for means* [37] has been adopted. In the proposed iterative design, a new replication is carried out in each iteration, what eventually decreases the variance of the parameters. Thus, new simulations are performed until the computed confidence intervals satisfy the parameters.

The simulation algorithm used to perform the simulations (both for the Welch’s procedure and step 2) is the *exact stochastic simulation algorithm* proposed by Gillespie [39]. Once a new replication is performed, it is resampled at equal sampling intervals by applying a linear interpolation (step 3). The sampling interval is the same for all replications, and it is set to the average time interval between events of the first replication.

The average coordinates referred in steps 4 and 6 are computed just on the steady state, i.e., the transient state determined in the first step is disregarded. Once the origin (*a*_*x*_,*a*_*y*_) of the polar coordinates is obtained (step 4), the transformation to polar coordinates can be carried out (step 5). Finally, the average *ρ* and polar angular speed *ξ* are computed (step 6), and the confidence intervals for *ρ* and *ξ* can be calculated (step 7).

*ODE in polar coordinates.* A similar approach to the one discussed above can be taken to describe the system dynamics in terms of an ODE in polar coordinates. According to the following ODE, the system evolution is characterized by its expected changes in distance and angle to a reference point *a*:

(11) $$  {\fontsize{9.5}{6}\begin{aligned} \frac{d\rho}{dt}&=R(\mathbf{X}) \mathbb{E}\left[\Delta f_{\rho}(\mathbf{X})\right] =\sum\limits_{j=1}^{n} W_{j}(\mathbf{X}) f_{\rho}(\mathbf{X}{+}\nu^{j})-R(\mathbf{X}) f_{\rho}(\mathbf{X}) \\ \frac{d\psi}{dt}&=R(\mathbf{X}) \mathbb{E}\left[\Delta f_{\psi}(\mathbf{X})\right] =\sum\limits_{j=1}^{n} W_{j}(\mathbf{X}) \left(\,f_{\phi}(\mathbf{X}{+}\nu^{j})\right.\\&\left.\quad+\, g(\mathbf{X},\nu^{j},a)\right) {-}R(\mathbf{X})f_{\phi}(\mathbf{X}) \end{aligned}}  $$

where **X**=(*X*,*Y*) are the cartesian coordinates, (*ρ*,*ψ*) are the polar coordinates with origin at (*a*_*x*_,*a*_*y*_), *f*_*ρ*_(**X**) and *f*_*ψ*_(**X**) are defined as $f_{\rho }(\textbf {X})=\sqrt {(X-a_{x})^{2}+(Y-a_{y})^{2}}$ and *f*_*ψ*_(**X**)=atan(*Y*−*a*_*y*_,*X*−*a*_*x*_), and:

$$  g(\mathbf{X},\nu^{j},a)= \left\{ \begin{array}{ll} +2\pi & \text{if}\,\, f_{\psi}(\mathbf{X}){>} {\pi}/{2} \,\, \text{and} \,\, f_{\psi}(\mathbf{X}{+}\nu^{j}) {<} {-}{\pi}/{2} \\ -2\pi & \text{if}\,\, f_{\psi}(\mathbf{X}){<} {-}{\pi}/{2} \,\, \text{and} \,\, f_{\psi}(\mathbf{X}{+}\nu^{j}) {>} {\pi}/{2} \\ 0 & \text{otherwise} \end{array} \right. $$

As in (4), the above ODE makes use of the expected increments of the coordinates and number of events per time unit to express the derivatives. The first(second) case of the above expression avoids the discontinuity of the angle returned by atan when the trajectory moves from the second to the third quadrant(from the third to the second quadrant). While the average of polar coordinates of replications can be used to estimate the mean circular motion, ODE (11) provides information of the instantaneous speed of change of the polar coordinates at each possible state of the system.

### Systems with more than two species

The previous subsection has discussed how stochastic oscillations can be detected in systems with two species, i.e., *q*=2, be means of polar coordinates. One might expect that for systems comprising more than two species, spherical coordinates for *q*=3, and hyperspherical coordinates for *q*>3 could be used. Nevertheless, these coordinates pose difficulties when trying to assess oscillations. Let us illustrate this by considering a system with three species. The state of the system at a given time can be expressed in the spherical coordinates (*ρ*,*θ*,*ϕ*), where *ρ* is the radial distance, *θ* is the polar angle, and *ϕ* is the azimuthal angle. The range of *θ* is usually restricted to the interval [0,*π*], and the range of *ϕ* to [0,2*π*].

**Fig. 4:**
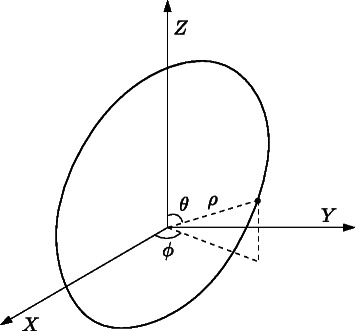
Assumed trajectory of a system with 3 species oscillating around a given reference point. As shown in Fig. 5 the time evolution of the polar angle *θ* exhibits no discontinuity for this trajectory

**Fig. 5:**
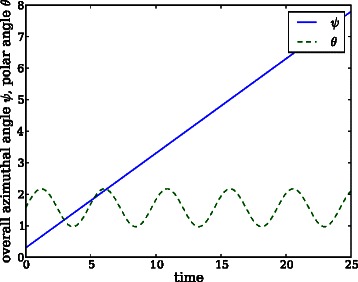
Potential time evolution of the overall azimuthal angle *ψ* and the polar angle *θ* of the oscillating system. In contrast to the angular coordinate in systems with two species, the polar angle in this trajectory presents no discontinuity. This fact hinders the use of spherical coordinates to assess oscillations in systems with more than two species

Assume that a system oscillates around a reference point which has been taken as the origin of the spherical coordinates, see Fig. 4. Assume further that the projection of the system trajectory on the plane *z*=0 moves anticlockwise if seen from a positive *z*. As the system trajectory evolves, the value of1469 *ϕ* increases until it reaches 2*π*. As in the previous subsection, in order to avoid discontinuities when the value of *ϕ* is close to 2*π* and 0, coordinate *ϕ* can be transformed to a new coordinate *ψ* that accounts for the overall angular distance run, see Fig. 5. By proceeding as in the previous subsection, one can average the value of this new coordinate over several replications, to estimate the system oscillations on the species associated to axes *X* and *Y*.

On the other hand, the value of the polar angle *θ* oscillates within the interval [0,*π*] and does not reach any of the limits of the interval, neither 0 nor *π*. Thus, there is no discontinuity in the polar angle to *fix*. Figure 5 shows the potential evolution of *θ* over time. Assume that several replications are performed, and the mean value of *θ* is estimated on the basis of its average value over the replications. As the system is stochastic, there will exist phase shifts among replications that will eventually involve a constant value of their average value. This renders the polar angle not useful to detect oscillations on the species associated to axis *Z*.

**Fig. 6:**
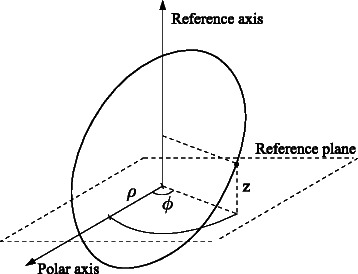
Cylindrical coordinates can be used to detect oscillations in systems with more than 2 species. According to this coordinate system, one cartesian coordinate and a tuple (*ρ*,*ϕ*) are used for systems with 3 species. In general, for systems with *q*>2 species, *q*−2 cartesian coordinates and a tuple (*ρ*,*ϕ*) are required. The choice of species associated to (*ρ*,*ϕ*) determines on which species the oscillation are to be assessed

A feasible way to overcome this difficulty is to consider cylindrical coordinates instead of spherical coordinates. To determine the position of a point, cylindrical coordinates establish a reference (or longitudinal) axis and a reference plane perpendicular to the axis, see Fig. 6. The origin is the intersection between the reference plane and the reference axis. The state of a system with three species is expressed in cylindrical coordinates as (*ρ*,*ϕ*,*z*), where (*ρ*,*ϕ*) are called polar coordinates (as they correspond to the polar coordinates on a plane parallel to the reference plane), and *z* is the height with respect to the reference plane.

In a system with 3 species (*a*,*b*,*c*), a natural choice is to associate one species, e.g. *c*, to the reference axis of the cylindrical coordinates. This way, the polar coordinates of (*a*,*b*) is (*ρ*,*ϕ*) which can be handled as in the previous subsection to avoid discontinuities, and estimate the stochastic oscillations on the populations of *a* and *b*. The same process can be repeated two more times: one with the reference axis associated to *a*, and one associated to *b*. This will uncover the oscillations on populations *b* and *c*, and *a* and *c* respectively. Thus, cylindrical coordinates allow us to *decouple* the oscillations between the different pairs of species involved in the system. Notice that, although the process is repeated 3 times for a system with 3, the same set of replications can be used in all three cases.

For systems with more than 3 species, a very similar approach can be taken. The height *z* in cylindrical coordinates can be interpreted as the *cartesian* coordinate of the system. If more species are involved, more cartesian coordinates must be considered, while just one tuple (*ρ*,*ϕ*) of polar coordinates must be considered. For instance, if the number of species is 5, three cartesian coordinates (*z*_1_,*z*_2_,*z*_3_) will be considered together with the polar coordinates (*ρ*,*ϕ*). The choice of species associated to (*ρ*,*ϕ*) determines on which species the oscillation are to be assessed.

*ODE in cylindrical coordinates.* The use of cylindrical coordinates enables us to use almost in a straightforward way the ODE (11) in polar coordinates presented in the previous subsection. For instance, the ODE in cylindrical coordinates for a system with 3 species **X**=(*X*,*Y*,*Z*) in which (*X*,*Y*) are associated to the polar coordinates and *Z* to the cartesian coordinate would become:

(12) $$  \begin{aligned} \frac{d\rho}{dt}&=R(\mathbf{X}) \mathbb{E}\left[\Delta f_{\rho}(\mathbf{X})\right] \\ \frac{d\psi}{dt}&=R(\mathbf{X}) \mathbb{E}\left[\Delta f_{\psi}(\mathbf{X})\right] \\ \frac{d Z}{dt}&=R(\mathbf{X}) \mathbb{E}\left[\Delta Z\right] \\ \end{aligned}  $$

where *f*_*ρ*_(**X**) and *f*_*ψ*_(**X**) are applied on the first two coordinates of **X**, i.e., *X* and *Y*. The above ODE accounts for the instantaneous changes of the polar coordinates of the two first species. An ODE for an arbitrary number of species can be easily derived from ODE (12).

**Parameters used in the case studies** The particular parameters used in the case studies reported in the Results and discussion section are the following: The *Welch’s procedure* makes use of 50 replications of the system, with a window of length 9, i.e., *w*=4, to smooth high frequency oscillations. The confidence intervals for the *replication/deletion approach for means* are specified with the parameters *α*=5 *%* and *M* *a* *x* *E* *r* *r*=3.

All the discussed methods have been developed in MATLAB [40]. The computations have been performed in a dual processor dual Core Intel Woodcrest (64 bits), with 2.33 Ghz and 4 GB RAM memory.

## Results and discussion

This sections applies the proposed method to assess oscillations to four case studies: an epidemic system, the Brusselator, a prey-predator system and the repressilator.

### An epidemic system

Consider an epidemic system [20] consisting of two species: susceptible and infected individuals; and five events: birth, death of a susceptible individual, contagion, recovery, and death of an infected individual. Let *S*=*X*_1_ and *I*=*X*_2_ be the number of susceptible and infected individuals, and *a*_*b*_=*W*_1_, *a*_*ds*_=*W*_2_, *a*_*c*_=*W*_3_, *a*_*r*_=*W*_4_ and *a*_*di*_=*W*_5_ be the transition rates of events birth, death of a susceptible individual, contagion, recovery, and death of an infected individual respectively. The system parameters are:

**System parameters:** *q* = 2, *n* = 5, $\nu =\left (\!\begin {array}{lccrr} 1&\, \, -1\, & -1\, &\, 1&\,\, 0\\ 0&\, \, 0& 1& -1\, & -1\end {array} \!\right)$, $a_{b}=\frac {S+I}{1+(b{\cdot }(S+I))/V}$, *a*_*ds*_=*m*_*S*_·*S*, $a_{c}=\beta \cdot S \cdot \frac {I}{V}$, *a*_*r*_=*r*·*I*, *a*_*dI*_=*m*_*I*_·*I*, *V*=5·10^3^, with *b*=0.4, *β*=10, *m*_*S*_=0.2, *m*_*I*_=5, *r*=3, and initial populations *S*(0)=4080 and *I*(0)=500.

**Fig. 7:**
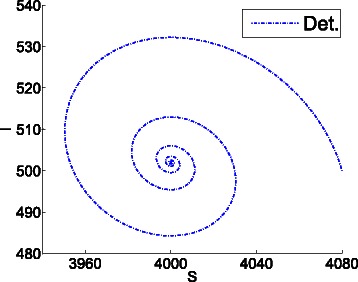
Deterministic trajectory of the epidemic system. This trajectory in the phase space is the solution of ODE (1), according to which the system reaches an equilibrium point at which both species, and hence the contagion rate, keep constant

Assume we are interested in evaluating the evolution of the contagion rate over time. Figure 7 shows the system evolution in the phase space according to ODE (1), the system reaches an equilibrium point at which both species, and hence the contagion rate, keep constant. The dotted line in Fig. 1 is the time evolution of the contagion rate according to this deterministic view. The solid line in Fig. 1 is the time evolution of the contagion rate according to a single stochastic replication of the Markov process. Unlike the deterministic evolution, the replication exhibits undamped oscillations with approximately constant frequency and amplitude. The Methods section (subsection *quadratic functions*) explores the deviation induced by ODE (1) with respect to the Markov process when estimating functions over the system trajectory.

**Fig. 8:**
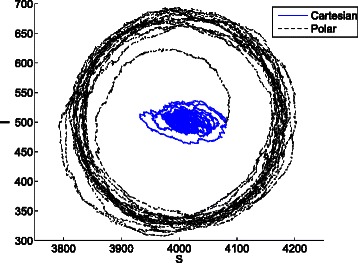
Average cartesian and polar coordinates of 66 replications of the epidemic system. The trajectory tending to the fixed point (4000,502) is the result of averaging the cartesian coordinates of the replications. The trajectory tending to a steady oscillation is obtained by averaging the polar coordinates as described in the Methods section. The average polar coordinates uncover the oscillating dynamics of the system at steady state

Figure 8 shows the trajectories in the phase space of 66 replications of the epidemic system. The trajectory tending to the fixed point (4000,502) is the result of averaging the cartesian coordinates of the replications, while the trajectory tending to a steady oscillation is obtained by averaging the polar coordinates as described in the Methods section. The interpretation of this figure is that the trajectories of the replications tend to loop around the fixed point.

The specified confidence intervals (see “Systems with more than two species” in the Methods section) were satisfied after 66 replications. At the steady state, the confidence interval for the average distance to the fixed point is *ρ*=176±5.217, and the interval for the angular speed is *ξ*=2.157±0.054. The time required to compute the replications was 1701 seconds.

**Fig. 9:**
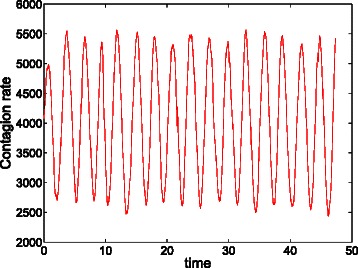
Contagion rate according to the average polar coordinates of 66 stochastic replications of the epidemic system. The average polar coordinates can be used to estimate values of interest as the contagion rate in the epidemic system. As expected, the time evolution of the contagion rate exhibits sustained oscillations

Informally, while the cartesian mean informs about the trajectory of the *center of mass* of the replications, the polar mean informs about the average *circular motion* of those replications. As the replications are stochastic, the averaged coordinates tend to steady state values. This results in the average cartesian coordinates tending to a constant value close to the fixed point, and the average polar coordinates tending to a circular motion with constant radius and constant angular speed. This way, the average polar coordinates uncover the oscillating dynamics of the system, what is accordance with the undamped oscillations shown in Fig. 1 for the contagion rate. Figure 9 shows the oscillating time evolution of the contagion rate according to the average polar coordinates.

### The Brusselator

The Brusselator is a theoretical model [41] for a type of autocatalytic reaction. The system consists of four reactions: *r*_1_:*A*→*X*, *r*_2_:2*X*+*Y*→3*X*, *r*_3_:*B*+*X*→*Y*+*D*, *r*_4_:*X*→*E*. The net reaction is *A*+*B*→*D*+*E* and the intermediate species are *X* and *Y*. As in other works [42], the populations of *A* and *B* will be kept constant to values *a* and *b* respectively, and the focus will be on the evolution of *X*=*X*_1_ and *Y*=*X*_2_. Under this assumption, the system parameters are the following:

**System parameters:** *q*=2,*n*=4, $\nu =\left (\!\!\begin {array}{lrrr} 1&\ \ 1& -1& -1\\ 0& -1&\ \ 1&\ \ 0 \end {array}\!\! \right)$, and the transition rates are *W*_1_(**X**)=*a*, *W*_2_(**X**)=*X* ^2^ *Y*/*V* ^2^, *W*_3_(**X**)=*b* *X*/*V*, *W*_4_(**X**)=*X*. Different volumes, initial populations and values of *a* and *b* will be considered.

The ODE (1) for this system is:

(13) $$  \begin{aligned} \frac{dX}{dt}&=a+X^{2}Y/V^{2}-bX/V-X\\ \frac{dY}{dt}&=bX/V-X^{2}Y/V^{2} \end{aligned}  $$

which has a fixed point at $\left (a,\frac {b}{a}V\right)$. This fixed point becomes unstable and ODE (1) exhibits a limit cycle when *b*>*V*+*a* ^2^/*V*.

**Fig. 10:**
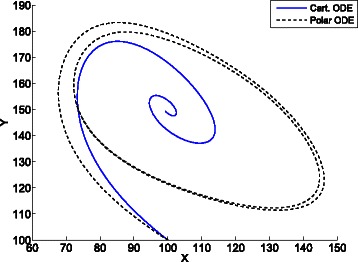
Trajectories of the cartesian ODE (1) and polar ODE (11) for the Brusselator. System parameters: *V*=100, *a*=100, *b*=150, *X*(0)=*Y*(0)=100. While the cartesian ODE (1) tends to the fixed point, the polar ODE (11), presents sustained oscillations that are also shown by the jump Markov process

**Fig. 11:**
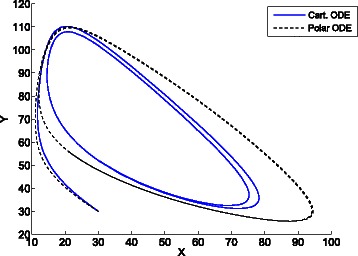
Trajectories of the cartesian ODE (1) and polar ODE (11) for the Brusselator. System parameters: *V*=30, *a*=1*V*, *b*=2.5*V*, *X*(0)=*Y*(0)=1*V*=30. The cartesian ODE (1)shows a limit cycle and scales with *V* (see Fig. 12)

**Fig. 12:**
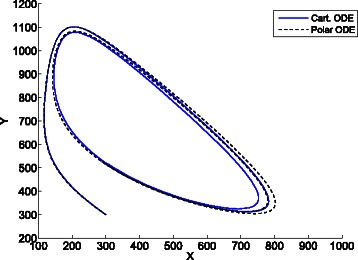
Trajectories of the cartesian ODE (1) and polar ODE (11) for the Brusselator. System parameters: *V*=300, *a*=1*V*, *b*=2.5*V*, *X*(0)=*Y*(0)=1*V*=300. Although the polar ODE (11) also enters a limit cycle for both sizes *V*=30 and *V*=300, it does not scale with *V* (see Fig. 11) and gets closer to the trajectory of the cartesian ODE for high values of *V*

Figures 10, 11 and 12 shows the different trajectories in the phase space yielded by the cartesian ODE (1), and the polar ODE (11). The trajectories in Fig. 10 correspond to parameters *V*=100, *a*=100, *b*=150, *X*(0)=*Y*(0)=100. It can be seen that while the cartesian ODE (1) tends to the fixed point *q*=(100,150), the polar ODE (11), which takes *q* as origin of the polar coordinates, presents sustained oscillations that are also exhibited by the jump Markov process.

Figures 11 and 12 show the trajectories of two models with same initial concentrations for all the species *X*(0)=*Y*(0)=1*V*, same values of parameters *a* and *b*, *a*=1*V*, *b*=2.5*V*, and different system sizes, *V*=30 and *V*=300 for Figs. 11 and 12 respectively. As expected, the cartesian ODE shows a limit cycle and scales with *V*. Although the polar ODE also enters a limit cycle for both sizes, it does not scale with *V* and gets closer to the trajectory of the cartesian ODE for higher values of *V*. In fact, in the limit *V*→*∞* the Markov process will converge to the cartesian ODE [15, 16], and then, the estimation of distances and angles will be the same both by the cartesian ODE and the proposed polar ODE, what will result in the same system trajectory.

**Fig. 13:**
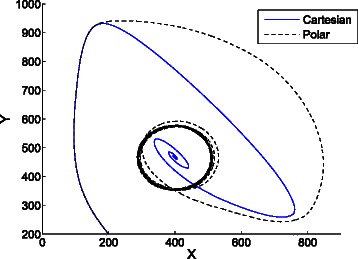
Average cartesian and polar coordinates of 1467 stochastic replications of the Brusselator with parameters *V*=30, *a*=1*V*, *b*=2.5*V*, *X*(0)=*Y*(0)=1*V*. While the cartesian average tends to an equilibrium, the polar average shows sustained oscillations

Figure 13 shows the trajectories in the phase space of the average cartesian and polar coordinates with parameters *V*=30, *a*=1*V*, *b*=2.5*V*, *X*(0)=*Y*(0)=1*V* for 1467 replications, which is the minimum number of replications to compute confidence intervals under the conditions specified in the Methods section. While the cartesian average tends to an equilibrium, the polar average shows sustained oscillations. The confidence interval for the average distance to the fixed point *q*=(100,150) is *ρ*=111.037±0.942, and the interval for the angular speed is *ξ*=307.923±9.238. The CPU time required for the computations was 14304 seconds.

### A prey-predator system

Let us consider a prey-predator model in which the number of preys is denoted by *X*_1_=*X*, and the number of predators by *X*_2_=*Y*. The system parameters are:

**System parameters:** *q*=2,*n*=4, $\nu =\left (\begin {array}{rclc} 1\ & -1\ & 0& 0\\ 0& 0& 1& -1 \end {array}\right)$, *W*_1_(**X**)=*α* *X*, *W*_2_(**X**)=*β* *X* *Y*/*V*, *W*_3_(**X**)=*δ* *X* *Y*/*V*, *W*_4_(**X**)=*γ* *Y* where *α*=10, *β*=0.01, *γ*=100, *δ*=0.02 and *V*=1.

The ODE (1) for this model are the well-known Lotka-Volterra equations [43]:

(14) $$  \begin{aligned} \frac{dX}{dt}&=X(\alpha-\beta Y)\\ \frac{dY}{dt}&=-Y(\gamma-\delta X) \end{aligned}  $$

The non-extinction fixed point is (*γ*/*δ*,*α*/*β*). The polar coordinates will take this fixed point as origin.

The squared distance from the system state (*X*,*Y*) to the fixed point *a*=(*γ*/*δ*,*α*/*β*) is given by *L*_*a*_(**X**)=(*X*−*γ*/*δ*)^2^+(*Y*−*α*/*β*)^2^. According to (8), the average speed of change of this squared distance is:

$$\begin{aligned} R(\mathbf{X})\mathbb{E}\left[\Delta L_{a}(\mathbf{X})\right] =&\, \sum\limits_{i=1}^{q} 2(X_{i}-a_{i})\left(\sum\limits_{j=1}^{n} {\nu_{i}^{j}}W_{j}(\textbf{X})\right)\\&+R(\textbf{X})\sum\limits_{i=1}^{q}\mathbb{E}\left[(\Delta X_{i})^{2}\right] \end{aligned} $$

For the given system parameters, it holds:

$$ \begin{aligned} \sum\limits_{i=1}^{q} 2(X_{i}-a_{i})&\left(\sum\limits_{j=1}^{n} {\nu_{i}^{j}}W_{j}(\mathbf{X})\right) \\ & = 2\left(X{-}\frac{\gamma}{\delta}\right)(\alpha X{-}\beta X Y/V)\\ &\quad+2\left(Y{-}\frac{\alpha}{\beta}\right)(\delta X Y/V{-}\gamma Y) \end{aligned} $$

and

$${\fontsize{7.3}{6} \begin{aligned} R(\mathbf{X})\sum\limits_{i=1}^{q}\mathbb{E}\left[(\Delta X_{i})^{2}\right] &=R(\mathbf{X})\left(\mathbb{E}\left[(\Delta X)^{2}\right]+\mathbb{E}\left[(\Delta Y)^{2}\right]\right) \\ & =R(\mathbf{X})\left(\sum\limits_{j=1}^{n} \left({\nu_{1}^{j}}\right)^{2}\frac{W_{j}(\mathbf{X})}{R(\mathbf{X})}+\sum\limits_{j=1}^{n} \left({\nu_{2}^{j}}\right)^{2}\frac{W_{j}(\textit{X})}{R(\textbf{X})} \right)\\ & = \bigl((1\cdot W_{1}(\mathbf{X})+ 1\cdot W_{2}(\mathbf{X}))+(1\cdot W_{3}(\mathbf{X})+1\cdot W_{4}(\mathbf{X}))\bigr) \\ & =\alpha X+\beta X Y/V+\delta X Y/V+\gamma Y \end{aligned}} $$

Then, by (8), the average speed of change of *L*_*a*_(**X**) becomes:

$$\begin{aligned} R(\mathbf{X})\mathbb{E}\left[\Delta L_{a}(\mathbf{X})\right] =&\, 2\left(X-\frac{\gamma}{\delta}\right)(\alpha X-\beta X Y/V)\\ &+2\left(Y{-}\frac{\alpha}{\beta}\right)(\delta X Y/V-\gamma Y)\\ &+ \alpha X+\beta X Y/V+\delta X Y/V+\gamma Y \end{aligned} $$

**Fig. 14:**
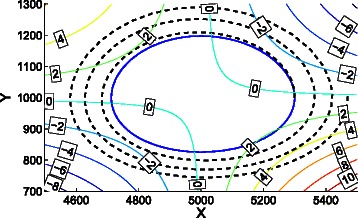
Trajectories of the cartesian ODE (1) and polar ODE (11) for the predator-prey system. The trajectory for the Lotka-Volterra equations, i.e., ODE (1), is the inner (solid) trajectory. The trajectory given by ODE (11) during 0.6 time units is the outer (dotted) trajectory. While the cartesian ODE produces a closed trajectory whose amplitude depends on the initial populations, the trajectory provided by the polar ODE moves away from the fixed point

Let the initial populations of the system be (5300,1000). The isolines shown in Fig. 14 correspond to the values of $R(\textbf {X})\mathbb {E}\left [\Delta L_{a}(\textbf {X})\right ]$, i.e., average speed of change of *L*_*a*_(**X**), divided by 10^6^. It can be observed that the system tends to move away from the fixed point $\left (\frac {\gamma }{\delta }, \frac {\alpha }{\beta }\right)=(5000,1000)$ since all the values of $R(\textbf {X})\mathbb {E}[\!\Delta L_{a}(\textbf {X})]$ around that point are positive.

The trajectory for the Lotka-Volterra equations, i.e., ODE (1), is the inner (solid) trajectory in Fig. 14. The trajectory given by ODE (11) during 0.6 time units is the outer (dotted) trajectory. While the cartesian ODE produces a closed trajectory whose amplitude depends on the initial populations, the trajectory provided by the polar ODE moves away from the fixed point, what is consistent with the *resonant stochastic amplification* and the tendency to extinction pointed out in [44] and [45]. As every replication eventually reaches extinction, no steady state exists and no average coordinates are computed.

**Fig. 15:**
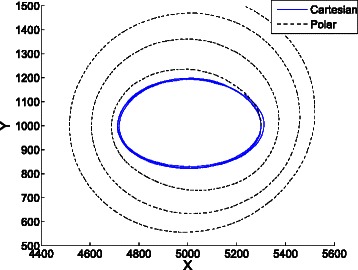
Average cartesian and polar coordinates of the predator-prey system. The trajectories are obtained as the average of 1000 stochastic replications of the predator-prey system. The cartesian average describes a closed orbit and the polar average tends to an extinction point

Figure 15 shows the average cartesian and polar coordinates of 1000 replications of the system during 0.65 time units. The computation time was 8825 seconds.

### The repressilator

The repressilator was proposed in [46] to show a stable oscillation which can be reported by the expression of green fluorescent protein. The model uses three transcriptional repressor systems that are not part of any natural biological clock. The model system was successfully induced in E. coli.

In this model, the repressor protein LacI (variable *X* denotes the mRNA and variable *PX* denotes the protein) inhibits the tetracycline-resistance transposon tetR (*Y*, *PY* denote mRNA and protein). Protein tetR inhibits the gene Cl from phage Lambda (*Z*, *PZ* denote mRNA and protein), and protein Cl inhibits lacI expression.

The data and parameters of the model are taken from the Biomodels database [47]. The system parameters can be found in Table 1, the constants derived from these parameters in Table 2, and the reactions and rates of the model in Table 3.

**Table 1:** System parameters of the repressilator model

Parameter	Value	Units
promotor strength (repressed) (*t* *p* *s*_*r* *e* *p* *r*)	5·10^−4^	*t* *r* *a* *n* *s* *c* *r* *i* *p* *t* *s*/(*p* *r* *o* *m* *o* *t* *o* *r*·*s*)
promotor strength (full) (*t* *p* *s*_*a* *c* *t* *i* *v* *e*)	0.5	*t* *r* *a* *n* *s* *c* *r* *i* *p* *t* *s*/(*p* *r* *o* *m* *o* *t* *o* *r*·*s*)
mRNA half life (*τ*_1/2,*mRNA*_)	2	*min*
protein half life (*τ*_1/2,*prot*_)	10	*min*
*K*_*M*_	40	*m* *o* *n* *o* *m* *e* *r* *s*/*c* *e* *l* *l*
translation efficiency (*eff*)	20	*p* *r* *o* *t* *e* *i* *n* *s*/*t* *r* *a* *n* *s* *c* *r* *i* *p* *t*
Hill coefficient (*n*)	2

**Table 2:** Constants of the repressilator model

Constant	Value	
average mRNA lifetime (*t*_*a* *v* *e*)	*τ*_1/2,*mRNA*_/*l* *n*(2)	=2.89 *m* *i* *n*
mRNA decay rate (*k* *d*_*m* *R* *N* *A*)	*l* *n*(2)/*τ*_1/2,*mRNA*_	=0.347 *m* *i* *n* ^−1^
protein decay rate (*k* *d*_*p* *r* *o* *t*):	*l* *n*(2)/*τ*_1/2,*prot*_	=0.0693 *m* *i* *n* ^−1^
transcription rate (*a*_*t* *r*):	*t* *p* *s*_*a* *c* *t* *i* *v* *e*·60	=29.97 *t* *r* *a* *n* *s* *c* *r* *i* *p* *t* *s*/*m* *i* *n*
transcription rate (repressed) (*a*0_*t* *r*):	*t* *p* *s*_*r* *e* *p* *r*·60	=0.03 *t* *r* *a* *n* *s* *c* *r* *i* *p* *t* *s*/*m* *i* *n*
translation rate (*k*_*t* *l*):	*e* *f* *f*·*k* *d*_*m* *R* *N* *A*	=6.93 *p* *r* *o* *t* *e* *i* *n* *s*/(*m* *R* *N* *A*·*m* *i* *n*)

**Table 3:** Reactions and rates of the repressilator model

No.	Reaction	Rate law
R1. Degradation of LacI transcripts	*X*→*∅*	*k* *d*_*m* *R* *N* *A*·*X*
R2. Degradation of TetR transcripts	*Y*→*∅*	*k* *d*_*m* *R* *N* *A*·*Y*
R3. Degradation of CI transcripts	*Z*→*∅*	*k* *d*_*m* *R* *N* *A*·*Z*
R4. Translation of LacI	*∅*→*P* *X*	*k*_*t* *l*·*X*
R5. Translation of TetR	*∅*→*P* *Y*	*k*_*t* *l*·*Y*
R6. Translation of CI	*∅*→*P* *Z*	*k*_*t* *l*·*Z*
R7. Degradation of LacI	*P* *X*→*∅*	*k* *d*_*p* *r* *o* *t*·*P* *X*
R8. Degradation of TetR	*P* *Y*→*∅*	*k* *d*_*p* *r* *o* *t*·*P* *Y*
R9. Degradation of CI	*P* *Z*→*∅*	*k* *d*_*p* *r* *o* *t*·*P* *Z*
R10. Transcription of LacI	*∅*→*X*	$a0\_tr+\frac {a\_tr\cdot KM^{n}}{KM^{n}+(PZ)^{n}}$
R11. Transcription of TetR	*∅*→*Y*	$a0\_tr+\frac {a\_tr\cdot KM^{n}}{KM^{n}+(PX)^{n}}$
R12. Transcription of CI	*∅*→*Z*	$a0\_tr+\frac {a\_tr\cdot KM^{n}}{KM^{n}+(PY)^{n}}$

**Fig. 16:**
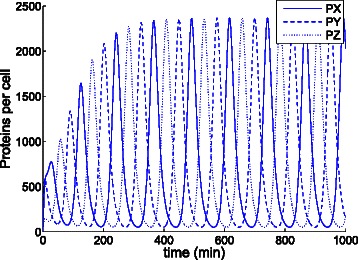
Evolution of the number of proteins of the repressilator model with *n*=2 according to the cartesian ODE (1). The steady state shows steady state oscillations in all three species

**Fig. 17:**
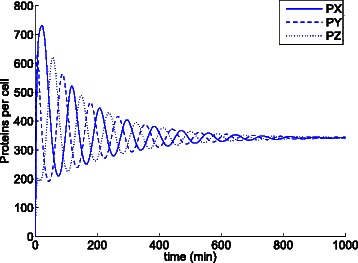
Evolution of the number of proteins of the repressilator model with *n*=1.5 according to the cartesian ODE (1). The system tends to a fixed point at which the populations of all three species remain constant

**Fig. 18:**
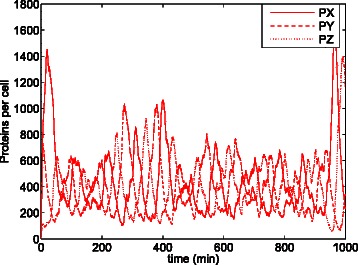
Evolution of the number of proteins of the repressilator model with *n*=1.5 according to one stochastic replication. In contrast to the steady state in Fig. 17, no constant steady state is reached. The existing oscillations cannot be considered random fluctuations since regular repetitive patterns, as peaks happening at regular time intervals, appear

**Fig. 19:**
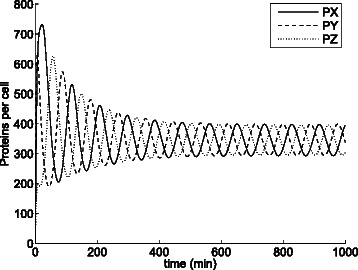
Evolution of the number of proteins of the repressilator model with *n*=1.5 according to the ODE in cylindrical coordinates (12). Unlike ODE (1) (see Fig. 17), ODE (12) captures the steady state oscillations of the stochastic system (see Fig. 18)

Figure 16 shows the trajectories over time yielded by cartesian ODE (1) for the original parameters of the model with initial populations *X*=*Z*=*P* *X*=*P* *Y*=*P* *Z*=0 and *Y*=20. If the Hill coefficient *n* is decreased from 2 to 1.5, the trajectories produced by ODE (1) do not show sustained oscillations and tend to a stable steady state (341.7,341.7,341.7), see Fig. 17. Nevertheless, a single stochastic replication, see Fig. 18, of the model reveals that oscillations are not damped as time passes. These sustained stochastic oscillations are easily captured by an ODE in cylindrical coordinates as the one in (12). Figure 19 shows the trajectories for the number of proteins yielded by an ODE in cylindrical coordinates where the coordinates for proteins PX and PY are handled as polar, and the rest of coordinates, i.e., PZ, X, Y and Z, are handled as cartesian, and the origin of the coordinate system is 341.7 for PX and PY, and 0 for the rest of species. Unlike the ODE in cartesian coordinates, the ODE in cylindrical coordinates exhibits undamped oscillations.

**Fig. 20:**
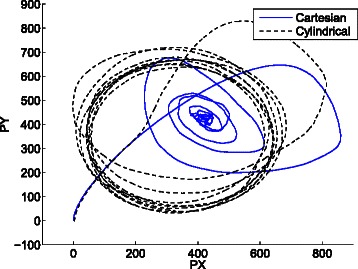
Trajectories of the average cartesian and cylindrical coordinates of 297 stochastic replications in the phase space (*P* *X*,*P* *Y*) of the repressilator model with *n*=1.5. While the average cartesian coordinates tend to a fixed point, the average cylindrical coordinates show sustained oscillations

Figure 20 shows the average cartesian and cylindrical coordinates in the projected phase space (*P* *X*,*P* *Y*) computed over 297 replications. After such number of replications, the parameters specified for the confidence intervals are satisfied. The confidence interval for the average distance to the reference point (*P* *X*=341.7,*P* *Y*=341.7) is *ρ*=307.964±9.22, and the interval for the angular speed is *ξ*=−0.0612±0.00095. Given the symmetry of the model, these confidence intervals also hold for cylindrical coordinates in which (*P* *X*,*P* *Z*) or (*P* *Y*,*P* *Z*) are taken as polar (more precisely, the only difference is that for (*P* *X*,*P* *Z*) the loops are clockwise, and therefore the angular speed is positive, i.e., *ξ*=0.0612±0.00095). The CPU time required for the computations was 5761 seconds.

## Conclusions

The population dynamics of many biological systems can be naturally modeled by means of jump Markov processes. The exact evolution of such processes is given by the Master Equation which can be solved only for very particular systems. Thus, alternative approaches must be considered to analyze the system dynamics.

This work has focused on evaluating the average steady state oscillations around a given reference axis. As these oscillations can be inherently stochastic and can be exhibited by systems with relatively low populations, they are often overlooked by methods relying on a deterministic fluid approximation of the Markov process, as the reaction rate equation. Thus, instead of relaxing or transforming the original stochastic description of the system, the proposed method deals directly with a set of stochastic replications.

The mean steady state values of the system dynamics can be computed by averaging the trajectories over time of the different replications. Traditionally, this average is computed separately on the time evolution of each species of the systems. Thus, if one considers the system trajectory in the phase space, this average corresponds to the average of the cartesian coordinates of the system. As it has been shown, this *cartesian perspective* can be myopic since the average trajectory of stochastically out of phase replications tends to flatten and does not show oscillations. However, other *perspectives* are possible allowing other points of view of the same stochastic system. The proposed method transforms the usual cartesian coordinates of pairs of species into polar coordinates, in which the angular coordinate accounts for the overall angular distance run by the system. This way, the oscillations are not canceled cancel out when the average polar coordinates are computed. The new coordinates can be straightforwardly used to obtain confidence intervals for the mean angular speed and distance to the reference axis.
